# Nervousness, Hereditary and Acquired

**Published:** 1886-01

**Authors:** E. Parsons

**Affiliations:** Savannah, Ga.


### Nervousness, Hereditary and Acquired.

BY E. PARSONS, D.D.S., SAVANNAH, GA.

Read before the Ohio State Dental Society, Chillicothe, October, 1885.

Every dentist of experience knows that one of our greatest troubles is the management of nervous patients. By better understanding the various phenomena of their physical condition, especially nervous manifestation, we may do much to quiet them by kind words and proper stimulation. If we fail to do this we lose their confidence without which it is very difficult to operate successfully. In general terms we divide the nervous system into two classes, the sensory and motor; although both are wisely provided by our maker for our good, they are often greatly abused. It is the opinion of many of our oldest physicians and dentists, that the abnormality of the nervous system of our people is on the increase. If this be so, the cause should be known and the laws of life obeyed. It is my purpose in this paper to present for your consideration some of the reasons why there is an increase of nervous affections of our people, affecting the whole system, especially the teeth.

Primary causation is often mysterious, and judged by its effects, which are often misleading, by reason of reflex action, facial neuralgia may be caused by a tooth even when perfectly sound. If we mistake the cause, we labor in vain to effect the cure desired. It is only by reading, observation and experience, that we are able to make a correct diagnosis of any given case; good and useful teeth have often been sacrificed for want of a better knowledge of the nervous system.

The nerves have their root in the brain, the projections from these ramify to every part of the body, giving sensation and motion. If one class of these are unduly exercised to the neglect of the other, it will make a nervous somebody, or a knownothing.

All the various animals below man are endowed with instinct and are unprogressive, the succeeding generation cannot learn anything from the past history of its race, their nervous constitution appears to remain the same from age to age, not being endowed with the faculty of rationality; their governing principle is impulse. Not so with man; man being endowed by his maker with the principles of liberty and reason can know the history of his race for ages past, and has capacity for endless progression, in all that constitutes true manhood. To realize the nervous degeneracy of our people, we must compare the present with the past, and note the different circumstances of both body and mind. In this paper I will consider my subject in the following order, viz: 1st, stolidity; 2d, nervous excitability; 3d, nervous sensitiveness; 4th, nervous depression or exhaustion.

First, nervous stolidity. Of this we can number only a few amongst all our patients. Much of their suffering is inherited from their parents, and they are taught to bear pain without a murmur; all such give us no trouble in performing any operation desired. As a rule our American Indians are of this class. As I wish to comment more at large on the influences of our modern civilization, I note thus briefly this division of the subject.

Second, nervous excitability. It is sufficient for my purpose to take the American revolution, to which we are indebted for the enjoyment of our free institutions, as a starting point from which there has been a gradual increasing nervous excitability of our people. The great advance in the arts and sciences of our day, is mainly due to the increasing nervous excitability of our people. It has manifested itself in almost every department of human industry. Where to-day would be our steamboats, railroads, telegraph, telephone and electric lights, and our own useful appliances, so highly prized in performing dental operations without it; compare our present status to what it was before these inventions, and if you can find a better reason for the establishment of our dental colleges and dental societies, raising our standard of excellence in every branch of our profession, thus quickening the intellect by reason of an increased nervous excitability, I shall be glad to know it.

Again, the nervous excitable people are more energetic than any other, and what they cannot do individually, they attempt by associated enterprise. Usually they suffer acutely from local irritation, and if a tooth be the cause they hasten to the dentist and willingly submit to anything that will give them speedy relief.

Third, nervous sensitiveness. By this I mean all those whose nerves are easily disturbed, both by mental and physical causes. A much larger proportion of my patients are of this class now than was the case forty years ago. In this short essay I can only enumerate some of the causes that have produced a marked and growing change in the nervous condition of our people. Amongst the most prominent, there are the three easy of observation :

1. Hereditary predisposition.
2. Education, domestic and public.
3. Reading exciting uovels.

First, hereditary predisposition. It is an old adage that "like begets like." Parents of a sharply defined nervous organization are usually very sensitive; their children are difficult for us to manage, and as population increases this class increases also. We often see children in the same family differing very widely in their nervous temperament. In this case you will find about the same difference between the father and mother. In my opinion, this noticeable fact can only be accounted for on the principle of magnetic phenomena, varying conditions at different times of conception and gestation.

Second, education. On this division of my subject I will briefly consider the moral, intellectual and physical training of children. If these three for an orderly, well developed character could be utilized harmoniously, both in domestic and public education, from infancy to maturity, we should soon have a far more vigorous population than we now have; but most unhappily this is not the case. The present system of education is very defective in this respect; the mother who for fear the child will soil its clothes, will not allow it to creep on the floor or play in the yard and treats it more like a doll baby, makes a great mistake, gives it little or no chance to develope its muscles and invigorate the whole nervous system, in this way children are generally cared for and treated, without the least regard to the laws of health, is it any wonder that so many die in infancy? The infant should receive its proper nourishment at regular stated times. Children should not be stuffed with all sorts of things. If children are sent to school before they are seven years old, the kindergarten system is the best now known or in use. By this system the children's whole course is to them one continued play, and they unconsciously acquire a love for useful knowledge and at the same time freely exercise the brain and muscles. Our public and private schools may satisfy the present state of public opinion, but they are managed without a due regard to the development of a good physical constitution. Every school should have a room or play ground with all suitable fixtures for gymnastic exercises together with moral and mental training or culture; this, although it would not increase their nervous sensitiveness, would prepare them to better bear the ills of life. This class of our patients usually have very sensitive teeth, and dread a visit to the dentist. They usually procrastinate until compelled by severe tooth-ache, and you all know that this is the worst time to operate satisfactorily. Again, I have noticed very delicate, sensitive mothers unconsciously impress upon their offspring, much of their nervous sensitiveness, and it is difficult to clean out a simple cavity without making them cry, which greatly interferes with the operation. My experience with children has forced me to believe that the present system of rearing and educating them is very defective, and can be greatly improved in developing true manhood. If a change of base is not made, the physical degeneration of our people is certain to continue.

Fourth, novel reading. The novels that punish vice and reward virtue, are useful in a social and moral point of view but those that unduly excite the imagination and the feelings, influence the passions, keep the whole system in a feverish condition, not only destroy a taste for useful knowledge, but are like many other forms of dissipation, destructive to a healthy condition of the nervous system, and contribute in no small degree to the increasing nervousness of our people. We all have the organ of mirth more or less developed, and when not indulged in to excess it is a healthy exercise for both body and mind.

Nervous depression or exhaustion. Among the many causes that produce depression of the nervous system, I will consider only two, namely, sickness and the abuse of the physical organs. I use the term sickness to designate both acute and chronic diseases. The one is slow and the other is quick in its work, of debilitating the whole nervous system.

The disease known as consumption is one of the most insidious our people are liable to, and carries many to a premature grave. The depression of the nerves is gradual, but unavoidable. Many dentists while operating, are not careful in the position of the body, cramp the abdominal muscles, which should always expand and contract freely with every breath while operating, or pain in the side will, as is often the case, be the result. The disease known as dyspepsia, so common amongst our people is a great depresser of the nerves; those afflicted with this disease are unable to digest sufficient food to keep the various organs of the body in a healthy state. Masturbation, in which so many of our youth indulge adds largely to the number of enfeebled, nervous constitutions. The abuse of the sexual organs has much evil effect.

It is no easy matter to operate on the teeth of our extremely sensitive, nervous patients. If the observation of one who has been fifty years in practice, is of any value to his loved professional brethren, they are welcome. The genius of the profession has been exerted to its utmost to find an obtunder that would in all cases produce the desired effect. I have experimented with all the articles said in our periodicals to be good obtunders, amongst the latestof which is cocaine, and others not in the books, and I find no article entitled to our confidence. The same article with some seems to be effective, while with others it has little or no effect. In some cases the inhaling a few breaths of nitrous oxide gas, or Mayo's vegetable vapor gas, will allow the operator to proceed with little or no pain. I have often found hard pressure on either the labial or lingual surface of the effected tooth will render the excavation of a cavity quite bearable.

It is natural that our nervous patients dread a dental operation. It is gratifying to them if we appear to sympathize with them, and we should use every means to quiet their fears except deceit, for if we deceive even a child, it will not believe anything we may say to it afterwards.

By knowing the cause of the downward progress in the degeneracy of our people, it is the duty of both physician and dentist to enlighten the public on the subject, and point out the means for improvement. If our food and drink does not contain sufficient of all or any of the chemical elements for building up and sustaining the tissues, they must be artificially supplied. The faulty structure in children's teeth is fearfully on the increase, but an efficient remedy is known and if persistently applied, will in time remedy the evil.

You are all doubtless acquainted with the different anæsthetics, used for the purpose of extracting teeth without pain, and they are proper subjects for discussion at any State dental society meetings. We all know that there are many who have not the courage to submit to the operation without an anesthetic. In my essay read at the last meeting of the Georgia State Dental Society, published in the *Dental Luminary*, August number, first and second pages, you may learn all I have to say about it, and I need not repeat here. Dental colleges lay the foundation, but associated effort, and our periodicals rear thereon a noble structure of scientific dentistry. Seek after truth ; possessing it we have power for good or ill.

Thanks for the kind invitation of your President to meet with you, but as I cannot be present, I send this my unworthy substitute.

